# Differential Modulation by Polystyrene Microplastics on the Toxic Effects of Pyrene and Its Derivatives in the Blue Mussel *Mytilus edulis*: Insights from Hemolymph Biomarkers

**DOI:** 10.3390/toxics14060505

**Published:** 2026-06-10

**Authors:** Rong Zhu, Shangchun Li, Yumiao Lu, Weifeng Chen, Miance Xie, Kaiping Xu, Xiaofeng Zhou

**Affiliations:** 1 Zhejiang Institute of Hydraulics and Estuary (Zhejiang Institute of Marine Planning and Design), Hangzhou 310020, China; 2 Zhejiang Key Laboratory of River-Lake Water Network Health Restoration, Hangzhou 310020, China; 3 Yangtze River Delta Estuarine Tidal Bore-Geomorphology-Ecology Observation and Research Station, Ministry of Water Resources, Beijing 100053, China; 4 School of Public Health, Southwest Medical University, Luzhou 646000, China; 5 College of Metrology Measurement and Instrument, China Jiliang University, Hangzhou 310018, China

**Keywords:** PAHs derivatives, microplastics, oxidative stress, *Mytilus edulis*

## Abstract

Polycyclic aromatic hydrocarbons (PAHs) in water are readily adsorbed onto microplastics, posing a combined threat to aquatic ecosystem safety. However, the role of microplastics in altering the toxicity of PAH derivatives remains largely unexplored. This study was conducted to investigate the individual and combined toxic effects of polystyrene (PS, 2 µm) microplastics with pyrene (Pyr) and its four derivatives, including 1-methylpyrene (Pyr–CH_3_), 1-hydroxypyrene (Pyr–OH), 1-aminopyrene (Pyr–NH_2_), and 1-pyrenecarboxylic acid (Pyr–COOH), on blue mussels (Mytilus edulis). After a seven-day exposure experiment, the variations in five biomarkers—superoxide dismutase (SOD), catalase (CAT), glutathione peroxidase (GPx), malondialdehyde (MDA), and acetylcholinesterase (AChE)—were measured in the hemolymph. Our results indicated a com-pound-specific toxicological profile: all derivatives exhibited higher toxicity than the parent Pyr. Specifically, Pyr–CH_3_ primarily induced oxidative stress, whereas Pyr–OH, Pyr–NH_2_, and Pyr–COOH mainly affected neuroregulatory function. More importantly, PS microplastics acted as a differential modulator under the mixture conditions: they exacerbated the neuroregulatory dis-turbance caused by parent Pyr but conversely alleviated the oxidative damage induced by all four derivatives. Notably, PS exacerbated the neuroregulatory disturbance induced by Pyr–CH_3_. These compound-specific interactions highlight that microplastics alter the toxic effects of organic pollutants, thereby modifying environmental risk profiles. Our findings provide new insights for the ecological risk assessment of PAHs in aquatic environments.

## 1. Introduction

Polycyclic aromatic hydrocarbons (PAHs), a class of environmental pollutants containing two or more fused aromatic rings [1], are widely distributed in water, soil, air, and organisms [2,3,4]. PAHs are considered to have carcinogenic and mutagenic toxicity due to their ability to indirectly induce DNA damage [5,6]. Numerous studies have demonstrated that PAH exposure adversely affects organismal growth, reproduction, and locomotor ability [7,8,9,10]. Moreover, PAHs dissolved in seawater can adsorb onto microplastics, forming PAH-enriched microplastics [11,12,13,14], and these microplastics may alter their toxicity to organisms [15].

Microplastics (1 μm–5 mm), as emerging pollutants, are widely distributed around the world and have been detected in oceans, freshwater, sediments, and even in the Arctic and Antarctica [16,17,18,19,20]. The presence of microplastics in the marine environment poses a great threat to the entire ecosystem. The sources, distribution characteristics, and toxicity to aquatic organisms of microplastic pollution have attracted considerable attention [19,21,22,23,24,25]. Several studies have demonstrated that microplastics can inhibit growth and reproduction, resulting in body lesions such as malformations, inflammation, and oxidative stress in aquatic animals [26,27,28,29,30]. Furthermore, owing to their high specific surface area and hydrophobicity, microplastics exhibit strong affinities for toxic organic compounds such as PAHs, efficiently accumulating these organic pollutants from the surrounding environment [31].

Research has shown that microplastics can act as vectors for PAHs in aquatic systems, transferring them to biota via ingestion and thereby threatening aquatic organism health [32]. Thus, understanding the toxicity of microplastic-adsorbed PAHs is crucial for assessing aquatic health risks. Studies have indicated that the presence of microplastics can enhance the carcinogenic risk of PAHs [33]. For example, co-exposure to microplastics and PAHs inhibited acetylcholinesterase (AChE) activity in juvenile goby fish (*Pomatoschistus microps*) [34]. However, in addition to parent PAHs, the environment contains a wide array of PAH derivatives [35]. These derivatives exhibit various functional groups (e.g., alkyl, hydroxyl, amino, carboxyl, halogen), making them structurally complex [36]. Such functional groups of PAHs may confer different biological effects on organisms compared with parent PAHs. For instance, 1-hydroxy-PAHs (PAHs-OH) have been reported to disrupt the endocrine and increase the health burden on humans [37,38,39]. However, research on the toxic effects of PAH derivatives on aquatic organisms, and whether these effects align with those of parent compounds, remains limited. Furthermore, a key safety question is whether microplastics uniformly amplify the risk of all PAH compounds, or whether they differentially modulate the hazard posed by parent molecules versus their transformed derivatives. Investigating these toxic effects is essential for evaluating the ecological risks associated with PAHs in the environment.

The blue mussel *Mytilus edulis* (Linnaeus, 1758) is widely perceived as a “model organism” because it can proportionally reflect contamination levels in the surrounding environment [40]. Blue mussels have been reported to ingest microplastics through filter feeding in natural environments [41,42,43]. Co-exposure to microplastics and PAHs has been shown to induce notable toxic responses in mussels, particularly oxidative stress at the physiological and cellular levels [44,45]. Hemolymph is the blood-plasma equivalent of the invertebrate circulatory system and plays a vital role in immune regulation and antioxidant defense [46]. Elucidating the antioxidant responses of hemolymph in mussels exposed to microplastics and PAHs can thus reveal early-warning signals of compromised organismal health and broader ecosystem risk. However, most studies have focused on the gills or digestive glands of mussels rather than the hemolymph [45,47,48]. Moreover, the combined toxicities of PAHs with different functional groups and microplastics have rarely been investigated in blue mussels. Polystyrene (PS), a widely distributed microplastic polymerized from styrene monomers containing numerous benzene rings, has been shown to adsorb various organic pollutants and act as a vehicle for PAHs to marine organisms [32,49,50]. Pyrene (Pyr), a PAH with four benzene rings, is also frequently used in studies on PAH toxicity and its adsorption interactions with microplastics [36,51,52,53,54]. The four selected derivatives (methyl-, hydroxyl-, carboxyl-, and amino- substituted PAHs) are highly representative model functional groups for PAH derivatives and have been widely employed as model compounds in the related literature [11,36,55].

Oxidative damage and neurotoxicity are commonly used to assess the toxic effects of PAHs [56,57,58]. Numerous biomarkers can reveal the damage intensity and indirectly reflect the degree of tissue peroxidation lesions and neurotoxicity. Superoxide dismutase (SOD), catalase (CAT), glutathione peroxidase (GPx), and malondialdehyde (MDA) are widely accepted oxidative stress biomarkers [57,58,59]. SOD is the foremost and most critical component of the antioxidant enzyme defense system against reactive oxygen species (ROS), particularly superoxide anion radicals [60,61]. CAT is one of the major antioxidant enzymes widely present in organisms. GPx is a critical component of the enzymatic antioxidant defense system that protects cells from oxidative stress, working with SOD and CAT. GPx exerts its antioxidant effect by catalyzing the reduction of H_2_O_2_ and organic hydroperoxides to water and the corresponding alcohols, respectively [62,63]. MDA, a stable metabolite of lipid peroxidation end products, reflects the potential antioxidant capacity and the rate of lipid peroxidation [64,65,66]. Acetylcholinesterase (AChE) is a critical neurotransmitter enzyme that catalyzes the hydrolysis of the neurotransmitter acetylcholine into choline and acetic acid in the synaptic gap of cholinergic synapses and neuromuscular junctions [67,68]. AChE is frequently selected as a neurotoxicity biomarker to investigate the effects of environmental pollutants [58,69,70]. In this study, the activity or concentrations of these biomarkers were measured to assess the extent of oxidative stress, thereby analyzing the tissue oxidative damage and toxic effects induced by PAHs.

Therefore, this study aimed to evaluate how PS microplastics modulate the sublethal toxicological risks of Pyr and four of its common derivatives (Pyr–CH_3_, Pyr–OH, Pyr–COOH, and Pyr–NH_2_) in the marine mussel *M. edulis*. Two critical scientific questions were addressed: (1) Do Pyr and its derivatives exhibit distinct toxicological profiles? (2) Do PS microplastics modulate the toxicity of Pyr and its derivatives, and if so, do these modulatory patterns differ among compounds? To answer these questions, a toxicological experiment was conducted in which mussels were exposed to Pyr or its derivatives for seven days, with or without PS. Five physiological-biochemical indices were measured to evaluate the effects on antioxidant defense, lipid peroxidation, and neurotoxicity. Our findings contribute to understanding how functional groups modify PAH toxicity in the presence of microplastics, enabling a more accurate assessment of PAH pollution risks in marine systems.

## 2. Materials and Methods

### 2.1. Materials Preparation

*M. edulis* specimens were collected from the Shengsi Marine Protected Areas in the East China Sea. After removing the attachments from the shell surface, the 2-year-old mussels were transported to the laboratory with seawater at 4 °C. Prior to the toxicity test, the mussels were cultured for one week in artificial seawater to adapt to the experimental environment. During the acclimation period, the culture water was renewed daily, and the mussels were regularly fed with *Chlorella vulgaris* [46]. Healthy individuals of similar size (wet weight: 55.8 ± 6.7 g, length: 8.1 ± 0.9 cm) were selected for the exposure experiments.

The artificial seawater (30‰ salinity) was prepared using commercial sea salt (Tropic Marin, Wartenberg, Germany). The sea salt was dissolved in ultrapure water and filtered through a 0.22 μm membrane to remove solid impurities. The artificial seawater was maintained at a salinity of 30 ± 1‰ and a pH of 8.1 ± 0.5. The culture temperature was 25 ± 1 °C, and the light condition was 12:12 h (light:dark) [46]. Salinity and pH of the artificial seawater were measured daily using a Yellow Springs Instrument (YSI) ProQuatro multiparameter meter (YSI Inc., Yellow Springs, OH, USA) before water renewal to ensure consistent culture conditions for the mussels.

Pyr was chosen as the representative PAH and used in this study. Pyr (≥99% purity) was purchased from Sigma-Aldrich (St. Louis, MO, USA). The Pyr derivatives, including 1-methylpyrene (Pyr–CH_3_, ≥97% purity), 1-hydroxypyrene (Pyr–OH, ≥98% purity), 1-carboxylpyrene (Pyr–COOH, ≥97% purity), and 1-aminopyrene (Pyr–NH_2_, ≥97% purity), were also purchased from Sigma-Aldrich (St. Louis, MO, USA). Pristine PS microplastics (2 μm diameter) were purchased from the Tianjin Baseline ChromTech Research Centre (Tianjin, China). The certified concentration of PS dispersed in pure water was 25 mg/mL, with a corresponding particle density was 5.61 × 10^9^ particles/mL.

### 2.2. PAHs and PS Exposure

The acclimated *M. edulis* were randomly divided into 12 experimental groups following a full factorial design of 6 PAH treatments (control, Pyr, Pyr–CH_3_, Pyr–OH, Pyr–COOH, and Pyr–NH_2_) × 2 PS microplastic treatments (with PS and without PS). Each group contained 3 replicate glass tanks (volume: 10 L), and each tank was stocked with 10 individuals under continuous aeration. The 12 treatment groups were as follows: (1) control; (2) Pyr only; (3) Pyr–CH_3_ only; (4) Pyr–OH only; (5) Pyr–COOH only; (6) Pyr–NH_2_ only; (7) control + PS; (8) Pyr + PS; (9) Pyr–CH_3_ + PS; (10) Pyr–OH + PS; (11) Pyr–COOH + PS; (12) Pyr–NH_2_ + PS. The exposure concentration of PS was 1.0 mg/L, referencing environmental concentrations (0.31–5.1 mg/L) in polluted aquatic environments [71] and previous toxicological test concentrations (3.1 mg/L) for mussels [72]. This concentration, which falls within the environmental range and lower than common toxicological doses, ensures ecological relevance and literature comparability. The exposure concentration of Pyr and its derivatives was 30 μg/L. This exposure concentration was referenced from previous studies [44,73,74,75,76]. Although the tested concentrations in this study may exceed environmentally realistic levels in most cases, this study was designed to investigate mechanistic biological responses, which can be more sensitively identified at relatively higher exposure concentrations.

The artificial seawater was renewed daily, and the mussels were regularly fed with *C. vulgaris* during the experimental period [46]. The corresponding PAHs and PS were supplemented to maintain consistent exposure concentrations. No mortality was observed during the experiment. After seven days of exposure, the mussels were sampled to assess antioxidant defense capacity, lipid peroxidation levels, and neurotoxicity.

### 2.3. Hemolymph Collection and Reserving

After 7 days of exposure, 4 mL of mussel hemolymph was extracted from the posterior adductor muscle using a sterile evacuated blood collection tube (5 mL). The hemolymph was then immediately centrifuged at 1000× *g* and 4 °C for 2 min (Eppendorf Centrifuge 5810 R, Eppendorf AG, Hamburg, Germany). The supernatant was removed, and the precipitation (haemocytes) was resuspended by pipette in 4 mL of 1× phosphate-buffered saline (PBS, pH 7.4) and used for analysis of the five physiological indicators. The hemolymph collection was modified based on the method described by Wang et al. [75].

### 2.4. Oxidative Stress and Neurotoxicity Assessment in Mussel Hemolymph

SOD activity was measured by detecting the inhibition of water-soluble tetrazolium salt-1 (WST-1) formazan formation at 450 nm according to the manufacturer’s instructions. CAT activity was determined by terminating the reaction with ammonium molybdate and measuring the resulting pale-yellow complex at 405 nm [77]. GPx activity was assessed by monitoring the rate of glutathione (GSH) consumption in the presence of H_2_O_2_. Residual GSH was reacted with 5,5′-dithiobis-(2-nitrobenzoic acid) (DTNB) to form 2-nitro-5-thiobenzoic acid (TNB), and the absorbance was then measured at 412 nm. MDA levels were quantified using the thiobarbituric acid (TBA) method, in which MDA reacts with TBA under high temperature and acidic conditions to form a pink adduct, and the absorbance was measured at 532 nm. AChE catalyzes the hydrolysis of acetylthiocholine to produce thiocholine, which reacts with DTNB to form yellow TNB. The absorbance was measured at 412 nm. All five biomarkers described above were measured using commercial kits purchased from Nanjing Jiancheng Bioengineering Institute (Nanjing, China). Protein content was determined using a commercial protein quantification kit from the same manufacturer, following the manufacturer’s protocol. All enzyme activities were calculated based on corresponding standard curves and normalized to total soluble protein content.

### 2.5. Statistical Analysis

Data for the five biomarkers were expressed as mean ± standard deviation (SD). Statistical analyses were performed using SPSS Statistics 24 (IBM Analytics, Armonk, NY, USA). Data normality and homogeneity of variance were tested prior to statistical analysis. All data conformed to a normal distribution and exhibited homogeneity of variance.

First, the PS-free exposure groups were analyzed separately. One-way analysis of variance (ANOVA) followed by Tukey’s HSD post-hoc test was used to compare the toxicity differences between parent Pyr and its derivatives. Subsequently, two-way ANOVA was employed to analyze the effects of parent Pyr and its derivatives, PS exposure, and their interaction on enzyme activities, thereby clarifying the modulatory effect of PS on the toxicity of Pyr and its derivatives. Differences were considered statistically significant at *p* < 0.05. Figures were created using Origin 8.0 (OriginLab Corporation, Northampton, MA, USA).

## 3. Results

### 3.1. Oxidative Stress and Neurotoxicity of Pyr or Pyr Derivatives on Hemolymph of Mussels

**Figure 1:**
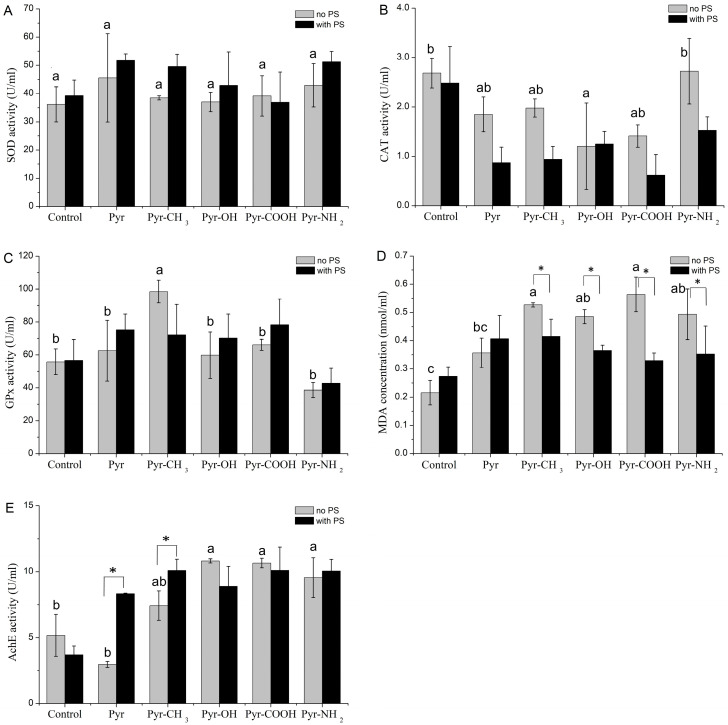
Oxidative stress indicators (SOD activity, (**A**); CAT activity, (**B**); GPx activity, (**C**); MDA concentration, (**D**)) and neurotoxicity indicator (AChE activity, (**E**)) in the hemolymph of mussels after 7 days of exposure to Pyr or its derivatives, with or without PS microplastics. Data are expressed as mean values ± SD (n = 3). Grey bars represent single-exposure groups (Pyr and its derivatives alone without PS), and black bars represent co-exposure groups (Pyr and its derivatives with PS). Different lowercase letters on all grey bars indicate significant differences among Pyr and its derivatives (one-way ANOVA, *p* < 0.05); asterisks (*) denote significant differences between single-exposure and co-exposure groups (two-way ANOVA, *p* < 0.05).

Antioxidant capacity and lipid peroxidation levels in the hemolymph of *M. edulis* were evaluated after a 7-day single or combined exposure to PS and PAHs. The oxidative stress indicators (SOD, CAT, GPx, MDA) and the neurotoxicity indicator (AChE) in the hemolymph of mussels exposed to Pyr and its derivatives showed different variations in Figure 1. One-way ANOVA was performed to compare toxicological differences between parent Pyr and its derivatives.

SOD activity in all experimental groups is presented in Figure 1A. No significant difference in SOD activity was observed among all groups, including the control, Pyr, and Pyr derivative treatments (F(5, 12) = 1.197, *p* = 0.367). This observation may be attributed to the fact that the basal antioxidant capacity of the mussels was sufficient to scavenge superoxide anions without inducing additional SOD synthesis, and that the exposure stress intensity did not reach the critical threshold for SOD activation [78,79].

CAT activity in all experimental groups is shown in Figure 1B. One-way ANOVA revealed a significant difference in CAT activity among the control, Pyr, and Pyr derivative groups (F(5, 12) = 4.779, *p* = 0.012). Post-hoc multiple comparisons indicated that CAT activity in the Pyr–OH treatment was significantly lower than that in the control group (*p* = 0.032). There was no significant difference in CAT activity between the Pyr exposure treatment and the Pyr derivative treatments.

GPx activity in the hemolymph of mussels from all experimental groups is presented in Figure 1C. The results indicated that GPx activity differed significantly among the control, Pyr, and Pyr derivative groups (F(5, 12) = 10.308, *p* = 0.001). GPx activity in the control group showed no significant difference from that of all treatment groups, except for the Pyr–CH_3_ treatment. GPx activity in thePyr–CH_3_ treament was significantly higher than that in the Pyr group (*p* = 0.013).

One-way ANOVA indicated that MDA levels among the control, Pyr, and Pyr derivatives were significantly different (F(5, 12) = 17.884, *p* < 0.001). MDA content in all Pyr derivatives treatments was obviously higher than that in the control group (Figure 1D). MDA content in the hemolymph of mussels exposed to Pyr–CH_3_ and Pyr–COOH was significantly higher than that in mussels exposed to Pyr (*p* = 0.020 and *p* = 0.005, respectively). No significant difference in MDA content was observed among the Pyr derivative treatments.

AChE activity in the hemolymph of mussels exposed to Pyr and its derivatives is shown in Figure 1E. Statistical analysis revealed a significant difference among the control, Pyr, and Pyr derivatives groups (F(5, 12) = 7.223, *p* = 0.002). There was no significant difference in AChE activity between the Pyr treatment group and the control group. In contrast, all Pyr derivative groups except Pyr–CH_3_ exhibited significantly elevated AChE activity compared with the Pyr groups (*p* < 0.05). AChE activity among the Pyr derivatives treatments showed no significant difference.

### 3.2. The Effect of PS on Oxidative Stress and Neurotoxicity in the Hemolymph of Mussels Exposed to Pyr or Pyr Derivatives

The variations in oxidative stress and neurotoxicity parameters in the hemolymph of mussels co-exposed to Pyr or its derivatives and PS are illustrated in Figure 1. Two-way ANOVA was performed to analyze the main effects of Pyr treatments and PS exposure, as well as their interactive effects.

SOD activity in the hemolymph of mussels from all treatments after a 7-day exposure experiment is presented in Figure 1A. Two-way ANOVA results revealed that the main effect of PS was not significant (F(1, 24) = 0.804, *p* = 0.379). The interaction effect between Pyr compounds and PS was also not significant (F(5, 24) = 0.324, *p* = 0.893), suggesting that the effect of PS on SOD activity was consistent across Pyr and its derivatives. Thus, PS exerted no significant effect on SOD activity in mussels exposed to Pyr or its derivatives.

CAT activity in the hemolymph of mussels exposed to Pyr and its derivatives, with or without PS, is shown in Figure 1B. The results revealed a significant main effect of PS on CAT activity (F(1, 24) = 20.526, *p* < 0.001), with PS exposure leading to decreased CAT levels compared with the non-PS condition. The Pyr compound × PS interaction was not significant (F(5, 24) = 1.784, *p* = 0.154), indicating that the modulatory effect of PS on CAT was consistent across Pyr and all its derivatives.

GPx activity of all treatments is presented in Figure 1C. The main effect of PS was not significant (F(1, 24) = 0.288, *p* = 0.597). The interaction effect between the Pyr compounds and PS was also not significant (F(5, 22) = 2.059, *p* = 0.109), suggesting that the effect of PS on GPx activity was consistent across Pyr and its derivatives. Accordingly, PS did not significantly alter GPx activity during exposure to Pyr or its derivatives.

Figure 1D presents MDA levels in the hemolymph of mussels exposed to Pyr and its derivatives, with or without PS. The main effect of Pyr and its derivatives on MDA was significant (F(5, 24) = 11.291, *p* < 0.001). The main effect of PS was also significant (F(1, 24) = 17.437, *p* < 0.001). Furthermore, the interaction effect between the Pyr derivatives and PS was significant (F(5, 24) = 5.352, *p* = 0.002), indicating that the effect of PS on lipid peroxidation was dependent on the specific Pyr derivative. Simple main effects analysis revealed that MDA levels in the control group and the parent Pyr group did not differ significantly between the PS-supplemented and PS-free groups (*p* = 0.237 and *p* = 0.309, respectively). By contrast, in all Pyr derivative groups, the presence of PS significantly reduced MDA concentrations compared with derivative-only exposures. These findings suggest that PS can alleviate the oxidative toxicity induced by Pyr derivatives.

AChE activity in all experimental groups, with or without PS, is shown in Figure 1E. The main effect of PS was not significant (F(1, 24) = 3.478, *p* = 0.074). Nevertheless, the interaction effect between the Pyr derivatives and PS was significant (F(5, 24) = 4.276, *p* = 0.006), indicating that the modulatory effect of PS on AChE activity was dependent on the specific Pyr derivative. Simple main effects analysis revealed that in the Pyr and Pyr–CH_3_ groups, AChE activity of the respective PS-supplemented group was significantly higher than that in PS-free group.

## 4. Discussion

### 4.1. Differentiated Toxicity Profiles of Pyr and Its Derivatives: Implications for Hazard Identification

The results revealed distinct oxidative stress profiles induced by Pyr and its derivatives, underscoring that the toxicity of PAHs is strongly correlated with their chemical structures, particularly the types of substituted functional groups. These findings are consistent with previous studies indicating that PAH derivatives generally exhibit greater toxicity than their parent compounds in aquatic systems [80,81]. Among all tested derivatives, Pyr–CH_3_ induced the most pronounced oxidative stress. Compared with the parent Pyr and other derivatives treatments, the Pyr–CH_3_ group showed markedly elevated GPx activity accompanied by a significant increase in MDA levels, suggesting that methyl substitution enhances the pro-oxidant effect of Pyr [46]. This observation can be attributed to the intrinsic properties of the methyl group: as a hydrophobic electron-donating group, –CH_3_ increases the lipophilicity of Pyr (higher logP value), thereby promoting its accumulation in biological membranes and leading to elevated production of ROS. To counteract excessive ROS accumulation, organisms upregulate GPx expression to scavenge hydrogen peroxide and organic peroxides [55,62,63]. Notably, Pyr–CH_3_ did not significantly alter AChE activity, indicating that its toxic effects predominantly involve the oxidative stress pathway rather than neuroregulatory processes.

In contrast, Pyr–OH exerted a comparatively moderate effect on oxidative stress markers: GPx activity showed an increasing trend that did not reach statistical significance, and MDA levels were not significantly different from those in the Pyr group. However, this derivative significantly increased AChE activity, suggesting an alteration in neuroregulatory function. The introduction of a hydroxyl group (–OH) reduces the hydrophobicity of the compound (lower logP value), which may limit its passive transmembrane diffusion and consequently fail to elicit a strong GPx response [82]. Nevertheless, as a hydrogen bond donor, the hydroxyl group may interfere with neurotransmitter metabolism through hydrogen bonding or electrostatic interactions with the active site of AChE [8]. Furthermore, the phenolic hydroxyl group itself possesses free radical scavenging capacity, which may partially counteract lipid peroxidation [83,84].

The toxicity profile of Pyr–NH_2_ was similar to that of Pyr–OH: GPx and MDA levels were not significantly elevated, whereas AChE activity was significantly increased. The amino group (–NH_2_) also reduces the hydrophobicity of the compound and can act as a hydrogen bond donor [36,55], resulting in weaker oxidative stress but a marked alteration in neuroregulatory function. Moreover, –NH_2_ carries a positive charge, which may enhance its binding to the active site of AChE through electrostatic interactions, thereby interfering with neurotransmission [85].

Pyr–COOH exhibited a unique toxicity profile: despite its low hydrophobicity, it significantly increased MDA content and AChE activity. The –COOH group, which is partially deprotonated and negatively charged at physiological pH, might be taken up via anion transporters (a hypothesis requiring experimental verification), leading to intracellular accumulation and lipid peroxidation [86]. Its strong polarity also enhances AChE binding, potentially disrupting neuroregulation [85,87]. Thus, polar derivatives may exert toxicity mainly through transporter-mediated uptake rather than passive diffusion [88]; however, direct evidence (e.g., transporter inhibition assays) is required to confirm this speculation.

Previous studies have demonstrated that exposure to PAHs leads to inhibition of AChE [47,56,89,90]. In contrast, our results revealed that AChE activity in the hemolymph of mussels exposed to Pyr derivatives was significantly higher than that in the parent Pyr and the control groups. These findings suggest that PAH derivatives can alter AChE activity, thereby disturbing cholinergic homeostasis and potentially affecting neuroregulatory function. Moreover, increases in AChE activities have also been observed in studies focusing on toxic effects of exposure to various pollutants [91,92]. These discrepancies may be attributed to multiple factors, such as exposure regimens and tissue specificity [93]. We propose that the observed AChE elevation is primarily a derivative-specific over-activation effect rather than a compensatory response. The polar substituents of Pyr derivatives enhance the aryl hydrocarbon receptor (AhR)-mediated signaling pathway, induce more intense oxidative stress via the ROS/nuclear factor erythroid 2-related factor 2 (Nrf2) pathway, and directly upregulate the expression and activity of AChE [94,95]. Our findings demonstrate that PAH derivatives exhibit significantly stronger toxic effects compared with their parent compounds. These results are consistent with those of Freitas et al. [37], who found that OH-PAHs exacerbated the pathological progression of acute myocardial infarction. Research on zebrafish has also shown that OH-PAHs produce higher toxicity than the parent PAH [96], and that nitro-PAHs are more carcinogenic and mutagenic than PAHs [97,98].

### 4.2. PS Differentially Modulates the Toxicity of Pyr and Its Derivatives

The presence of PS differentially modulated the toxicity of Pyr and its derivatives, with the modulation pattern clearly dependent on the functional group [99]. For the parent Pyr, PS selectively affected neuroregulatory function (elevated AChE activity) without affecting oxidative stress markers (SOD, CAT, GPx, and MDA), suggesting that partial adsorption onto PS followed by endocytic uptake increases intracellular target exposure. In contrast, the reduction in free Pyr concentration was insufficient to mitigate oxidative damage [8,36]. For the hydrophobic methyl derivative Pyr–CH_3_, PS induced a dual response: it significantly reduced MDA content (alleviated oxidative stress) while increasing AChE activity (enhanced neuroregulatory disturbance). This observation supports the hypothesis that extensive sorption of Pyr–CH_3_ onto PS surfaces lowers the free compound concentration, thereby relieving oxidative stress [36]. Only freely dissolved PAHs are significantly bioavailable and toxic to aquatic organisms [100]. However, it is possible that internalization of PS–compound complexes via endocytosis leads to increased total intracellular exposure, which in turn enhances neurotoxicity, possibly due to the targeting of internalized complexes to mitochondria rather than causing widespread oxidative stress [101,102]. Similar “Trojan horse” effects have been suggested as a plausible mechanism for other hydrophobic pollutants in the presence of PS [103]. Consistent with this hypothesis, literature evidence indicates that PS microplastics might act as vectors, facilitating the transfer of PAHs to marine bivalves and potentially resulting in their bioaccumulation within tissues [11,45]. For instance, Avio et al. [44] found that the PS-adsorbed Pyr could effectively deliver the contaminant load to mussel leading to bioaccumulation. We emphasize that these interpretations remain hypotheses based on indirect evidence and require direct experimental verification in future studies.

For the three polar derivatives (Pyr–OH, Pyr–NH_2_, and Pyr–COOH), PS significantly reduced MDA levels and alleviated oxidative damage in mussels. The polar groups exhibit low sorption affinity for PS [11,36], so the modulation is minimal and largely reflects a non-specific antioxidant effect of PS itself, possibly through free radical scavenging or interference with lipid peroxidation chain reactions. This pattern is consistent with other studies showing that microplastics can reduce certain oxidative stress markers without altering other endpoints [48].

In the broader context of microplastic–PAH interactions, conflicting reports exist. For instance, co-exposure of mussels to PS and fluoranthene (Flu) increased histopathological damage and antioxidant marker levels [45], and synergistic effects on oxidative stress, lipid peroxidation, and DNA damage have been observed in bivalves [8]. However, other studies using polyethylene microplastics with Flu found no obvious additive effects [57,74], and fish exposure experiments showed that microplastics did not amplify the acute toxicity of PAHs [104]. These discrepancies may arise from differences in microplastic type, size, experimental species, and exposure duration [105,106,107,108]. The presence of PS affects the toxicity of Pyr and its derivatives primarily through two competing mechanisms: enhanced cellular uptake of PS-adsorbed compounds via endocytosis versus reduced free concentration due to strong sorption. The balance between these two mechanisms depends on the functional-group-dependent affinity of each compound for PS and its intrinsic membrane permeability. Our results highlight a critical and underappreciated aspect of environmental safety: microplastics may inadvertently sequester certain emerging contaminants, locally reducing their immediate biological risk while creating a reservoir of adsorbed pollutants.

Although the proposed mechanism—that differential adsorption onto PS modulates the bioavailability and hence the toxicity of Pyr versus its derivatives—is supported primarily by indirect evidence and literature, our toxicological endpoint data (e.g., MDA reduction in derivative + PS groups) are consistent with this conclusion and align with established sorption principles [11,36]. Direct experimental verification was beyond the scope of this work. The exposure duration in this study was only 7 days, which may not truly reflect long-term chronic toxic effects, and the relatively limited replication (n = 3) may not fully capture biological response variability. The actual aqueous concentrations of PAHs and water quality parameters were not measured. This may introduce uncertainties in interpreting microplastic–PAH interaction effects, as water chemistry strongly influences their bioavailability. Therefore, future studies are warranted to conduct longer-term experiments with increased replicates, including simultaneous measurement of PAH aqueous concentrations and water quality parameters. Furthermore, further investigations incorporating simultaneous measurements of adsorption isotherms for each compound onto PS under identical conditions, coupled with quantitative analysis of PAH bioaccumulation in mussel tissues (e.g., digestive gland), would be essential to conclusively establish the causal link between PS-mediated sorption, internal dose, and the observed biological effects. The toxicity mechanisms of PAH derivatives, as well as the mechanisms underlying the effects of PS on PAHs, require verification through additional experimental studies. Nevertheless, our findings provide a strong phenotypic foundation for this mechanistic framework and highlight the critical need to consider pollutant-specific interactions with microplastics in risk assessments.

## 5. Conclusions

In this study, the toxic effects of Pyr and its four derivatives (Pyr–CH_3_, Pyr–OH, Pyr–NH_2_, and Pyr–COOH) on the hemolymph of *M. edulis* were evaluated in the absence and presence of PS microplastics, with oxidative stress markers and neurotoxicity endpoints measured. The results showed that all derivatives exhibited higher toxicity than the parent Pyr. Specifically, Pyr–CH_3_ primarily induced oxidative stress, whereas the other Pyr derivatives mainly affected neuroregulatory function. The presence of PS differentially modulated the toxicity in a functional-group-dependent manner: PS selectively exacerbated the disruption of neuroregulatory function induced by the parent Pyr; for Pyr–CH_3_, PS significantly alleviated oxidative stress but enhanced the disturbance of neuroregulatory function; for the three polar derivatives (Pyr–OH, Pyr–NH_2_, and Pyr–COOH), PS significantly reduced MDA levels, indicating alleviated oxidative damage. These findings provide a scientific basis for more comprehensive ecological risk assessment of PAHs in aquatic environments and also carry important practical implications for the mitigation of marine environmental pollution.

## Data Availability

The raw data supporting the conclusions of this article will be made available by the authors on request.
